# Uropathogens Antimicrobial Sensitivity and Resistance Pattern From Outpatients in Balochistan, Pakistan

**DOI:** 10.7759/cureus.17527

**Published:** 2021-08-28

**Authors:** Taimoor Hussain, Mehdi Moqadasi, Sheza Malik, Asjad Salman Zahid, Kefayatullah Nazary, Shafi M Khosa, Mohammad Mohsin Arshad, John Joyce, Rajeswari Khan, Sneha Puvvada, Khalida Walizada, Abdul Rahim Khan

**Affiliations:** 1 Neurology/General Practitioner, Bolan Medical College, Quetta, PAK; 2 Medical Laboratory Technology, Shafa Khana Sahib Zaman Hosptial, Quetta, PAK; 3 Medicine, Army Medical College Rawalpindi, Rawalpindi, PAK; 4 Internal Medicine, Allama Iqbal Memorial Teaching Hospital, Lahore, PAK; 5 Internal Medicine, Kabul University of Medical Science, Kabul, AFG; 6 Pathologist, Bolan Medical College, Quetta, PAK; 7 Internal Medicine, Multan Medical and Dental College, Multan, PAK; 8 Intern, M.S. Ramaiah Medical College, Bangalore, IND; 9 Medicine and Surgery, College of Medicine & Sagore Dutta Hospital, Kolkata, IND; 10 Medicine and Surgery, M.S. Ramaiah Medical College, Bangalore, IND; 11 Neurological Surgery, Ali Abad Teaching Hospital, Kabul, AFG; 12 Internal Medicine, Jinnah Medical and Dental College, Karachi, PAK

**Keywords:** uropathogens, antimicrobial resistance, antibiotic resistance, urinary tract infections, quetta, pakistan, antimicrobial surveillance, uti

## Abstract

Objective

To determine the pattern of microbes responsible for urinary tract infections and their susceptibility to different antibiotics.

Method

This is a cross-sectional study conducted at Quetta, Pakistan. The urine samples of 400 patients were collected and sent for culture and sensitivity analysis. The results were recorded on an excel datasheet. Descriptive statistics were used to describe the data.

Results

Out of 400 urine samples, 266 samples were culture positive for microorganisms. The most common organism on analysis was *Escherichia coli* 123/266 (46.24%) followed by *Staphylococcus saprophyticus* 59/266 (22.18%) and *Klebsiella pneumonia* 49/266 (18.42%). Gram-negative microorganisms were most susceptible to fosfomycin, cefoperazone/sulbactam, and meropenem. Gram-positive microorganisms were most susceptible to fosfomycin, cefoperazone/sulbactam, meropenem, and amoxicillin/clavulanate. High rates of resistance in *E. coli* were observed to most commonly prescribed broad-spectrum antibiotics; ceftriaxone (64.35%), cefotaxime (76.54%), ceftazidime (49.43%), cefepime (53.44%), levofloxacin (71.26%), and amoxicillin/clavulanate (70.31%). *E. coli* was the major multidrug-resistant organism.

Conclusion

High rates of antibiotic resistance and multi-drug resistance were revealed in this study due to the widespread and injudicious use of broad-spectrum antibiotics. Thus, it is highly recommended to regulate the pharmacies. Physicians should judiciously prescribe antibiotics and practice the culture and sensitivity of urine samples rather than blind prescription. Continued surveillance on uropathogens prevalence and resistance, new and next-generation antibiotics, and rapid diagnostic tests to differentiate viral from bacterial infections is the need of time.

## Introduction

Urinary tract infections (UTIs) are among the most common types of infectious disease, accounting for approximately 150-250 million cases globally per year [1]. They are usually caused by gram-negative enteric rods, such as *Escherichia coli (E. coli), Klebsiella, Proteus, *etc. [2]. Approximately 50% of women acquire a UTI at least once in their lifetime [2]. The incidence of UTI among children is reported to be 30% all over the world [3]. 27% of women have a confirmed recurrence within the next six months, after the first episode of UTI [4]. UTIs can lead to renal scarring, ultimately leading to end-stage renal disease, therefore early diagnosis and treatment of UTI are necessary [5].

Antimicrobial resistance (AMR) is widespread across the globe, from the Americas to Australasia [6-16]. According to estimates by the Center for Disease Control (CDC), antibiotic-resistant bacteria cause at least 2.8 million illnesses and 35,000 deaths in the United States alone annually [17]. A study of trends of AMR in Europe observed that gram-negative uropathogens had high resistance to some of the most common antimicrobials. A north-to-south gradient in AMR exists in Europe, with higher resistance among southern European states like Greece, Cyprus, France, and Italy [18]. In a study of AMR in Asia pacific, "reduced sensitivity to commonly prescribed advanced-generation cephalosporin, piperacillin-tazobactam, and levofloxacin, among the studied gram-negative pathogens - *Enterococcus faecium, Staphylococcus aureus, Klebsiella pneumoniae, Acinetobacter baumannii, Pseudomonas aeruginosa, Enterobacter spp *(ESKAPE) were observed” [16]. One of the causes of antibiotic resistance is the empirical treatment of UTI [9-10]. In India, resistant bacterial infections resulted in 58,000 infants deaths in 2013 [19]. In the first and second years of life, infants and toddlers spend around 40 days on antibiotics, which clearly illustrates antibiotic overuse [20]. 85% of children with acute otitis media, below two years of age, are prescribed antibiotics [21]. Around 500 million courses of antibiotics are used to treat both bacterial and viral diarrhea each year across four middle-income countries India, Indonesia, Nigeria, and Brazil [10]. Nevertheless, antibiotic abuse for a diarrheal disease is a leading cause of increasing antibiotic resistance (ABR) [22]. Therefore, the regional antibiotic susceptibility patterns of the microorganisms should be used for treatment. World Health Organization's global plan of action on AMR has stressed AMR surveillance across nations as an important strategy for countering AMR [23]. This study is one of the few addressing antibiotic susceptibility and resistance patterns in Baluchistan, Pakistan. More extensive AMR surveillance data is the need of time.

## Materials and methods

This is a cross-sectional study conducted at Quetta, Pakistan over a period of nine months. The sampling technique was non-probability consecutive sampling. Informed consent was taken from all the participants. The study was conducted in the outpatient department of the hospital. The inclusion criterion was patients presenting with symptoms of uncomplicated urinary tract infection. The exclusion criteria of the study were patients who had refused to participate, immunocompromised, patients suffering from phimosis or paraphimosis, uncircumcised males, and patients who had taken antibiotics within the past 24 hours. The urine samples of the patients were then taken to confirm the diagnosis. Early morning mid-stream samples of urine were collected in sterile containers and immediately processed for further procedures. Urine samples with significant bacterial growth ≥ 10^5 ^CFU/mL were considered positive. Colony study and biochemical tests were done to identify the microorganisms. MacConkey agar (Oxoid, England) was used to subculture the colonies to get pure growth of the microorganisms. Biochemical tests were done to identify the microorganisms. Tests performed for Gram-positive cocci include catalase, coagulase, and novobiocin test. Tests performed for gram-negative bacilli include Simmon's citrate agar, methyl red, urease, sulfur indole motility test, and triple sugar iron test. Kirby-Bauer disk diffusion method was used to determine the antibiotic susceptibility of the isolated colonies. Müller-Hinton agar plates were used to identify the sensitivity pattern. After this, the measurement of the zone of inhibition of bacterial growth was performed and a comparison was done with the guidelines of the Clinical and Laboratory Standards Institute (CLSI, 2018).

The organisms were subjected to various groups of antibiotics including penicillins, fluoroquinolones, aminoglycosides, cephalosporins, and tetracyclines. In addition, based on previous urine culture studies in Pakistan, due to high rates of resistance to recommended antibiotics, fosfomycin, meropenem, and vancomycin were also tested. The culture and sensitivity results for the specimens were recorded on a google form and an excel sheet was produced. The excel data sheet was analyzed for sensitivity and resistance patterns of microorganisms. Descriptive statistics were used to describe the data in terms of numbers and percentages

## Results

Out of 400 urine samples, 266 samples were culture positive for microorganisms. 85 culture-positive samples were obtained from male participants and 181 culture-positive samples from female participants. *E. coli* was the most frequently observed microorganism followed by *Staphylococcus saprophyticus* and* Klebsiella pneumoniae*. The details of the frequency of uropathogens in urinary samples are shown in Table 1.

**Table 1:** Frequency of uropathogens in urinary samples.

Organism (n=266)	Frequency (%)
E. coli	123 (46.24)
Klebsiella pneumonia	49 (18.42)
Proteus mirabilis	13 (4.88)
Staphylococcus saprophyticus	59 (22.18)
Staphylococcus epidermidis	22 (8.27)

*E. coli* was the most common organism in both genders. The second most common organisms were *Klebsiella pneumoniae *in males and *Staphylococcus saprophyticus* in females. The frequency distribution of bacteria based on gender is depicted in Table 2.

**Table 2:** Division of uropathogens according to gender.

Uropathogens	E. coli (n=123)	Klebsiella pneumonia (n=49)	Proteus mirabilis (n=13)	Staphylococcus saprophyticus (n=59)	Staphylococcus epidermidis (n=22)
Male (85)	44 (51.76)	20 (25.88)	4 (4.7)	9 (10.58)	8 (9.41)
Female (181)	79 (43.64)	29 (16)	9 (4.97)	50 (27.62)	14 (7.73)

Gram-negative bacteria were more common than gram-positive in males (78.82%) and females (64.64%). Table 3 depicts the microorganism’s gram stain distribution pattern based on gender.

**Table 3:** Microorganisms gram stain distribution pattern based on gender.

Gender	Frequency No. (%)	Gram-Positive	Gram-Negative
Male	85 (31.9)	18 (21.17)	67 (78.82)
Female	181 (68.04)	64 (35.35)	117 (64.64)

High rates of resistance were observed to the most commonly prescribed antibiotics. *E. coli* were resistant to cephalosporins such as ceftriaxone (70.07%), cefepime (57.7%), and ceftazidime (48.7%). Cefepime resistance was more than 50 % in all organisms except *Staphylococcus saprophyticus* which showed comparatively lower resistance (27.77%). *E. coli* resistance pattern to fluoroquinolones was ciprofloxacin (67.4 %) and levofloxacin (71.5 %). *E. coli* showed high resistance to aminoglycoside gentamycin (58.5%) and lower resistance to amikacin (27.6%). Amoxicillin/Clavulanate also had high rates of resistance in all microorganisms except *Staphylococcus saprophyticus* which showed better sensitivity (70%). Vancomycin which is the therapy of choice for serious staphylococcal infections when penicillin and cephalosporin are resistant or cannot be used had high rates of resistance against *Staphylococcus saprophyticus* (86.2%) and *Staphylococcus epidermidis* (72.5%).* E. coli* showed good sensitivity to antibiotics such as cefoperazone/sulbactam (93.4%), fosfomycin (85.3 %), and meropenem (85.3 %). Sensitivity and resistance pattern to various groups of antibiotics was tested. The details of sensitivity and resistance patterns are depicted in Table 4.

**Table 4:** Uropathogens antibiotic sensitivity and resistance patterns S = Sensitive; R = Resistant; IS = Intermediate sensitive

Antibiotic	Bacteria/ Patterns	E. coli (n=123)	Klebsiella pneumonia (n=49)	Proteus mirabilis (n=13)	Staphylococcus saprophyticus (n=59)	Staphylococcus epidermidis (n=22)
Fosfomycin	Tested on	123	49	13	59	22
	S	105 (85.3%)	39 (79.59%)	8 (61.53%)	35 (59.32%)	12 (54.54%)
	R	12 (9.75%)	9 (18.36%)	5 (38.46%)	22 (37.28%)	8 (36.36%)
	I	6 (4.87%)	1 (2.04%)	-	2 (3.38%)	2 (9.09%)
Ceftriaxone	Tested on	101	40	12	53	21
	S	33 (32.67%)	17 (42.5%)	3 (25%)	20 (37.73%)	12 (57.14%)
	R	65 (64.35%)	19 (47.5%)	8 (66.66%)	25 (47.16%)	7 (33.33%)
	I	3 (2.97%)	4 (10%)	1 (8.33%)	8 (15.09%)	2 (9.52%)
Cefotaxime	Tested on	81	37	5	50	13
	S	19 (23.45%)	16 (43.24%)	3 (60%)	23 (46%)	1 (7.69$)
	R	62 (76.54%)	21 (56.75%)	2 (40%)	25 (50%)	11 (84.61%)
	I	-	-	-	2 (4%)	1 (7.69$)
Ceftazidime	Tested on	89	39	9	42	14
	S	42 (47.19%)	30 (76.92%)	5 (55.55%)	15 (35.71%)	5 (35.71%)
	R	44 (49.43%)	9 (23.07%)	4 (44.44%)	25 (59.52%)	9 (64.28%)
	I	3 (3.37%)	-	-	2 (4.76%)	-
Cefepime	Tested on	58.	21	6	18	11
	S	23 (39.65%)	11 (52.38%)	1 (16.66%)	12 (66.66%)	4 (36.36%)
	R	31 (53.44%)	10 (47.61%)	3 (50%)	5 (27.77%)	7 (63.63%)
	I	4 (6.89%)	-	2 (33.33%)	1 (5.55%)	-
Amoxicillin	Tested on	18	16	2	22	10
	S	1 (5.55%)	0 (0%)	-	7 (31.81%)	3 (30%)
	R	17 (94.44%)	16 (100%)	2 (100%)	15 (68.18%)	7 (70%)
	I	-	-	-	-	-
Amoxicilin/Clavulanate	Tested on	64	36	4	30	7
	S	17 (26.56%)	15 (41.66%)	-	21 (70%)	4 (57.14%)
	R	45 (70.31%)	20 (55.55%)	4 (100%)	9 (30%)	3 (42.85%)
	I	2 (3.12%)	1 (2.77%)	-	-	-
Ofloxacin	Tested on	67	37	4	35	8
	S	20 (29.85%)	16 (43.24%)	1 (25%)	16 (45.71%)	2 (25%)
	R	45 (67.16%)	21 (56.75%)	3 (75%)	19 (54.28%)	6 (75%)
	I	2 (2.98%)	-	-	-	-
Ciprofloxacin	Tested on	94	30	10	41	14
	S	21 (22.34%)	11 (36.66%)	2 (20%)	18 (43.90%)	4 (28.57%)
	R	64 (68.08%)	17 (56.66%)	8 (80%)	23 (56.09%)	9 (64.28%)
	I	9 (9.57%)	2 (6.66%)	-	-	1 (7.14%)
Levofloxacin	Tested on	87	40	10	36	17
	S	24 (27.58%)	16 (40%)	3 (30%)	18 (50%)	4 (23.52)
	R	62 (71.26%)	23 (57.5%)	6 (60%)	18 (50%)	13 (76.47%)
	I	1 (1.14%)	1 (2.5%)	1 (10%)	-	-
Norfloxacin	Tested on	83	37	12	40	13
	S	25 (30.12%)	12 (32.43%)	4 (33.33%)	9 (22.5%)	2 (15.38%)
	R	58 (69.87%)	25 (67.56%)	8 (66.66%)	30 (75%)	11 (84.61%)
	I	-	-	-	1 (2.5%)	-
Cefoperazone/Sulbactam	Tested on	60	38	4	24	9
	S	56 (93.33%)	35 (92.10%)	4 (100%)	22 (91.66%)	9 (100%)
	R	4 (6.66%)	3 (7.89%)	-	1 (4.16%)	-
	I	-	-	-	1 (4.16%)	-
Meropenem	Tested on	62	24	5	23	9
	S	52 (83.87%)	23 (95.83%)	2 (40%)	13 (56.52%)	7 (77.77%)
	R	5 (8.06%)	1 (4.16%)	-	6 (26.08%)	2 (22.22%)
	I	4 (6.45%)	-	3 (60%)	4 (117.39%)	-
Vancomycin	Tested on	Not tested	Not tested	Not tested	29	14
	S	-	-	-	2 (6.89%)	1 (7.14%)
	R	-	-	-	25 (86.20%)	11 (78.57%)
	I	-	-	-	2 (6.89%)	2 (14.28%)
Gentamycin	Tested on	54	13	6	25	14
	S	21 (38.88%)	5 (38.46%)	2 (33.33%)	11 (44%)	6 (42.85%)
	R	28 (51.85%)	7 (53.84%)	4 (66.66%)	11 (44%)	7 (50%)
	I	5 (9.25%)	1 (7.69%)	-	3 (12%)	1 (7.14%)
Amikacin	Tested on	58	17	8	28	16
	S	30 (51.74%)	9 (52.94%)	3 (37.5%)	20 (71.42%)	9 (56.25%)
	R	14 (24.13%)	7 (41.17%)	4 (50%)	7 (25%)	5 (31.25%)
	I	14 (24.13%)	1 (5.88%)	1 (12.5%)	1 (3.57%)	2 (12.5%)
Tetracycline	Tested on	67	37	3	36	15
	S	13 (19.40%)	18 (48.64%)	1 (33.33%)	12 (33.33%)	5 (33.33%)
	R	54 (80.59%)	19 (51.35%)	2 (66.66%)	24 (66.66%)	10 (66.66%)
	I	-	-	-	-	-
Doxycycline	Tested on	33	26	1	19	4
	S	13 (39.39%)	15 (57.69%)	-	15 (78.94%)	2 (50%)
	R	19 (57.57%)	11 (42.30%)	1 (100%)	4 (21.05%)	2( 50%)
	I	1 (3.03%)	-	-	-	-

The multidrug-resistant (MDR) pattern was also explored. Some of the samples were resistant to more than one antibiotic of the same or different groups. *E. coli *was the major multidrug-resistant organism, MDR in *E. coli* ranged from 1.6-17% while in *Staphylococcus saprophyticus* it ranged from 1.7-18.64%. Thirty-eight (14.28%) out of the 266 samples were multidrug-resistant to cefotaxime, ceftazidime, and ceftriaxone. 35 (13.15%) samples were multidrug-resistant to cefotaxime, ceftazidime, and ciprofloxacin. Twenty-six (9.77%) samples were multidrug-resistant to amoxicillin/clavulanate, ceftriaxone, and levofloxacin. Seventeen (6.3%) samples were multidrug-resistant to gentamycin, ciprofloxacin, and ceftriaxone. 15 (5.6%) samples were resistant to amoxicillin/clavulanate, ceftriaxone, and doxycycline. Eight (3%) samples were multidrug-resistant to four antibiotics, amoxicillin/clavulanate, levofloxacin, ceftriaxone, and doxycycline. Four (1.5%) of urine samples were resistant to amoxicillin/clavulanate, gentamycin, ciprofloxacin, ceftriaxone. Three (1.1%) urine samples were resistant to gentamycin, ceftriaxone, ciprofloxacin, and fosfomycin. Table 5 depicts the findings.

**Table 5:** Multi drug resistance (MDR) patterns of uropathogens The percentages are shown in parenthesis. The percentage of total MDR to a specific combination of antibiotics is calculated out of 266 culture-positive samples. The percentage MDR for a specified microorganism against a specified combination of antibiotics is calculated based on their relative frequencies CTX = Cefotaxime; CPZ = Ceftazidime; CRO = Ceftriaxone; CPE = Cefepime; LVO = Levofloxacin; CPR = Ciprofloxacin; AMC = Amoxicillin/Clavulanate; GEN = Gentamycin; FOS = Fosfomycin; DOX = Doxycyline; MPM = Meropenem.

		Gram-negative uropathogens			Gram-positive uropathogens	
Antibiotic Combinations	Total	E. coli, n (%)	Klebsiella pneumonia, n (%)	Proteus mirabilis, n (%)	Staphylococcus Saprophyticus, n (%)	Staphylococcus epidermidis, n (%)
AMC, GEN, FOS	2 (0.7)	0	0	2 (15.38)	0	0
AMC, GEN,CPR	6 (2.2)	3 (2.4)	1 (20.4)	1 (7.6)	1 (1.7)	0
AMC, CRO, LVO	26 (9.7)	19 (15.44)	5 (10.2)	0	1 (1.7)	1 (4.5)
AMC, CRO, LVO, MPM	2 (0.7)	2 (1.6)	0-	0-	0-	0-
AMC, CRO, DOX	15 (5.6)	6 (4.8)	5 (10.2)	1 (7.6)	2 (3.3)	1(4.5)
AMC, LVO, GEN	7 (2.6)	3 (2.4)	1 (20.4)	1 (7.6)	1 (1.7)	1 (4.5)
AMC, LVO, CRO, DOX	8 (3)	5 (4)	3 (6.2)	0-	0-	1 (4.5)
AMC,GEN,CPR,CTX	4 (1.5)	2 (1.6)	1 (20.4)	0	1 (1.7)	0
AMC, GEN, CPR, FOS	1 (0.3)	0	0	1 (7.6)	0	0
AMC, GEN,CPR,CTX,CPZ	4 (1.5)	2 (1.6)	1 (20.4)	0	1 (1.7)	0
AMC, GEN,CPR,CTX,CPZ,CRO	0	0	0	0	0	0
AMC,GEN,CPR,CTX,CPZ, CRO, LVO	0	0	0	0	0	0
GEN,CPR,CTX	17 (6.3)	12 (9.7)	1 (20.4)	0	4 (6.7)	0
GEN,CPR,CTX , FOS	3 (1.1)	1 (0.8)	0	0	2 (3.3)	0
GEN,CPR,CTX,CPZ	13 (4.8)	6 (4.8)	3 (6.2)	0	4 (6.7)	0
GEN,CPR,CTX,CPZ,CRO	8 (3.0)	4 (3.2)	1 (20.4)	0	3 (5)	0
GEN,CPR,CTX,CPZ,CRO, LVO	3 (1.1)	2 (1.6)	1 (20.4)	0	0	0
CPR,CTX,CPZ,CRO	27 (10.1)	17 (13.8)	3 (6.2)	1 (7.6)	6 (10)	0
CTX,CPZ,CRO	38 (14.3)	20 (16.26)	3 (6.2)	1 (7.6)	11 (18.64)	3 (13.63)
CPR,CTX,CPZ	35 (13.1)	21 (17.07)	5 (10.2)	1 (7.6)	8 (13.55)	0
CPR,CTX,CPZ, LVO	25 (9.3)	14 (11.38)	5 (10.2)	1 (7.6)	5 (8.4)	0
CRO,CTX,CPZ,CPE	13 (4.8)	7 (5.6)	2 (4)	0	1 (1.7)	3 (13.63)

## Discussion

UTIs are caused by several microorganisms. *E. coli* (46.2%) followed by *coagulase-negative staphylococci (CONS) *(30.6%) and *Klebsiella pneumoniae *(18.4 %) were the most common uropathogens in our study. A brief review of the most common uropathogens isolated from urine culture studies in Pakistan is as follows. Various urine culture studies in Pakistan have reported *E. coli* as the most common uropathogen ranging from 52%-77.4 % [24-29]. Another common organism was *Klebsiella pneumoniae* ranging from 1.9%-18.69% [24-29]. Few of the other uropathogens reported were *Pseudomonas aeruginosa, Enterobacter, and Proteus mirabilis* [24-29]

After the discovery of penicillin, antibiotics have revolutionized healthcare. No doubt, it has played a significant role in decreasing the mortality rate of various infections. Unfortunately, its widespread and indiscriminate use has led to antibiotic resistance especially in countries with poor regulation of the health sector. As per our study, the rate of resistance of *E. coli* to the most commonly prescribed antibiotics was as follows; ceftriaxone (64.35%), cefotaxime (76.54%), ceftazidime (49.43%), cefepime (53.44%), levofloxacin (71.26%), and amoxicillin/clavulanate (70.31%). A similarly high rate of resistance is observed in* CONS, Klebsiella pneumonia, and Proteus mirabilis* species, to most commonly prescribed antibiotics, as presented in Table 4. As per our study, *E. coli* showed the highest sensitivity to cefoperazone/sulbactam (93.3%), followed by fosfomycin (85.36 %), and meropenem (83.87%). Klebsiella Pneumonia showed the highest sensitivity to meropenem (95.83%), followed by cefoperazone/sulbactam (92.10%), fosfomycin (79.59%), and ceftazidime (76.92%). *Staphylococcus saprophyticus *showed the highest sensitivity to cefoperazone/sulbactam (91.66 %), followed by doxycycline (78.94%), amikacin (71.42%), and amoxicillin/clavulanate (70%). *Staphylococcus epidermidis* showed the highest sensitivity to cefoperazone/sulbactam (100%) followed by meropenem (77.77%), amoxicillin/clavulanate, and ceftriaxone (57.14%) each.

Alarming rates of antibiotic resistance have been observed by other researchers in Pakistan. A brief description of such studies is reviewed here. Farooqi et al. observed that in the seven years study period from 1990-1997, the microorganisms were observed to have a low rate of resistance to cefotaxime, ceftriaxone, aztreonam, ofloxacin, amikacin, gentamicin, and nitrofurantoin, despite an increase in antibiotic resistance [24]. However, antibiotic resistance has increased now to the most commonly prescribed antibiotics as observed by more recent studies. According to a study by Muzamil et al., “almost more than 60% of the total sample were resistant to co-amoxiclav, cefotaxime, and ceftriaxone, piperacillin/tazobactam, cefoperazone/sulbactam, and ciprofloxacin. All 100% cultures were resistant to amoxicillin” [25]. Their findings are similar to our study except for cefoperazone/sulbactam which showed better sensitivity in our study. Malik et al. concluded that “the rates of resistance of amoxicillin/clavulanate, ciprofloxacin, ceftriaxone, ceftazidime were almost 70 %. The cultures were more than 90% sensitive to amikacin, fosfomycin, and nitrofurantoin. Imipenem showed a sensitivity of 85%” [26]. The rates of resistance and sensitivities in their study are similar to ours except for nitrofurantoin which was not tested in our study . In their study Rizvi et al. observed that “more than 75% of the strains were resistant to fluoroquinolones. 63% of *E. coli* samples were found resistant to ceftriaxone and cefepime. However, the resistance profile towards nitrofurantoin, fosfomycin, and carbapenem was less than 10.7%.” [27]. In their study, Inam Ullah Khan et al. reported “The susceptibility pattern of *E. coli* showed that approximately more than 80% of the bacterial isolates were sensitive to imipenem, amikacin, tazobactam/piperacillin, and 69% to nitrofurantoin. Sensitivity to commonly used oral antibiotics was very low” [28]. Basharat Ali Khan et al. observed that “Out of 246 patients (20.43%) with nosocomial urinary tract infections, almost more than 60% of the samples were resistant to cefotaxime, ciprofloxacin, and gentamycin [29].

All the aforementioned studies indicate rising antibiotic resistance to some of the most commonly prescribed and broad-spectrum antibiotics. The high level of resistance is alarming when compared to developed countries like America. The prevalence of extended-spectrum beta-lactamase producers among the Enterobacteriaceae was less than 10% in North America, much lower as compared to Asia and the Middle East countries (>40%), according to “Study for Monitoring Antimicrobial Resistance Trends (SMART)” [30]. Health authorities, physicians, and pharmacies should work together to tackle this problem, regulate the healthcare industry especially prescription and purchase of antibiotics. Anyone in Pakistan can self-prescribe antibiotics and pharmacies dispense them without any prescription. Self-medication’s overall prevalence was reported to be 39% in a review of 34 studies from low-middle-income countries with 31,340 participants, [31]. The short duration of treatment, insufficient doses, taking the wrong medicine and inappropriate sharing of medicines were some of the most common malpractices [31]. On the other hand, doctors also overprescribe antibiotics, without following standard treatment algorithms. America’s national strategy to combat antibiotic resistance is an exemplary initiative for other countries. It recommends that “Improved detection can be achieved through appropriate data sharing, expansion, and coordination of existing surveillance systems, and establishment of a standardized platform for resistance testing and genetic characterization of bacteria. Development of Rapid “point-of-need” tests to distinguish between viral and bacterial infections and identifying bacterial drug sensitivities, new and next-generation antibiotics, diagnostics, and vaccines is the need of time. Rapid detection and control of outbreaks is another strategy to combat AMR. Global collaboration and capacities should be enhanced” [32].

Despite all efforts, certain limitations exist in the study. First, the urine culture and sensitivity data are from a single institution with smaller sample size, and can not be generalized to all UTI patients. Secondly, the data is primarily focused on urine culture findings, therefore, epidemiologic and demographic characteristics and comorbid conditions of patients are not extensively described.

## Conclusions

High rates of antibiotic resistance and multi-drug resistance were revealed in this study due to the widespread and injudicious use of broad-spectrum antibiotics. Thus, it is highly recommended to regulate the pharmacies. Physicians should judiciously prescribe antibiotics and practice culture and sensitivity of urine samples rather than blind prescription. Efforts to increase the global AMR surveillance research should be increased. New generation antibiotics, advanced "point-of-need" diagnostics to differentiate bacterial and viral infections, and vaccines should be developed.
